# The Role of Diet, Eating Behavior, and Nutrition Intervention in Seasonal Affective Disorder: A Systematic Review

**DOI:** 10.3389/fpsyg.2020.01451

**Published:** 2020-08-04

**Authors:** Yongde Yang, Sheng Zhang, Xianping Zhang, Yongjun Xu, Junrui Cheng, Xue Yang

**Affiliations:** ^1^Affiliated Wuhan Mental Health Center, Tongji Medical College of Huazhong University of Science and Technology, Wuhan, China; ^2^Wuhan East Body-Well Mental Hospital, Wuhan, China; ^3^Friedman School of Nutrition and Policy, Tufts University, Boston, MA, United States

**Keywords:** seasonal affective disorder, depression, diet, eating behavior, nutrition intervention, supplementation

## Abstract

**Background:** Seasonal affective disorder (SAD) is a biological and mood disorder with a seasonal pattern. Dietary intervention and nutritional status have been reported to affect SAD severity. The objective of this study was to systematically review the evidence of associations between SAD and diet, eating behavior, and nutrition intervention.

**Methods:** We performed a comprehensive search of MEDLINE, EMBASE, Web of Science, and Google Scholar from inception up to July 1, 2019. Studies that examined diet and eating behaviors in SAD patients and tests of nutrition interventions for SAD were included. Two independent investigators extracted data based on study designs, participants, outcomes, exposures, and association measures.

**Results:** Eleven studies were included: six studies examined distinctive dietary patterns and eating behaviors in SAD patients and five studies explored the efficacy of nutrition interventions for SAD. Vegetarianism and alcoholism were associated with higher SAD prevalence, but normal alcohol intake was not correlated with SAD severity. Compared with non-clinical subjects, SAD patients tended to consume significantly larger dinners and more evening snacks during weekdays and weekends and exhibit a higher frequency of binge eating, external eating, and emotional eating. Additionally, compared to healthy controls, SAD patients presented more cravings for starch-rich food and food with high fiber. However, neither the ingestion of carbohydrate-loaded meals nor Vitamin D/B12 supplementation showed benefit for SAD.

**Conclusion:** Studies suggest that SAD patients may exhibit distinctive diet preferences and eating behaviors, but no current nutrition intervention has demonstrated efficacy for ameliorating SAD symptoms. Further evidence is needed from randomized controlled trials with larger sample sizes and longer durations.

## Introduction

Seasonal affective disorder (SAD) is an almost-recurring annual depression with seasonality due to the alteration of mood, hormones, and gene expressions (Wirz-Justice, 2018). The onset of depression typically appears in autumn or winter with subsequent remission in spring or summer (Meesters et al., 2016). In the revised version of the Diagnostic and Statistical Manual of Mental Disorders III (DSM-III-R), the characteristics of SAD were first described by Rosenthal et al. as hypersomnia, overeating, and symptoms that respond to changes in climate and latitude (Rosenthal et al., 1984). The Seasonal Pattern Assessment Questionnaire (SPAQ) is frequently used as a self-administered screening tool for SAD in adult clinical and subclinical samples (Magnusson, 1996; Murray, 2003; Morales-Munoz et al., 2017). According to the DSM-5 guidelines, the diagnostic criteria of SAD include a history of over two depressive episodes and a seasonal pattern of depression in consecutive years (Dittmann et al., 1994; Battle, 2013).

A season pattern can be apparent in either major depressive disorder or bipolar disorder. Patients with bipolar disorder may exhibit symptoms of both depression and mania or hypomania, as opposed to recurrent depression (Gupta et al., 2013). SAD shows a regular progressive association between the onset of depression and the time of year and a full diminution of depressive symptoms at a certain point of the year. This distinguishes SAD from non-seasonal depression. Approximately 1–2% of the U.S. population experiences SAD annually, with symptoms present for around 40% of the year (Kurlansik and Ibay, 2012). Owing to its recurrence and long duration, SAD is considered a serious mental health disorder that imposes negative influences on patients' daily lives (Rohan et al., 2009). Light therapy, pharmacotherapy, and cognitive behavior therapy are common interventions for SAD patients. However, to this date, no single therapy or combination of therapies has been found to be superior (Kurlansik and Ibay, 2012).

Recent findings have shown that food or nutrition supplements and dietary patterns may affect the development of major depression disorders (Shipowick et al., 2009; Kerr et al., 2015; Deacon et al., 2017; Opie et al., 2018), while a deficiency of certain nutrients can be identified as predictors for depression (Chong et al., 2014). It has been reported that patients with depression symptoms tend to consume more carbohydrates and prefer night eating (Danilenko et al., 2008), leading to overeating and overweight (Rosenthal et al., 1984; Donofry et al., 2014). Accumulating evidence has shown that vitamin D deficiency is associated with the progression of depression (Shipowick et al., 2009; Gu et al., 2019). One systematic review with a meta-analysis of 31,424 participants revealed an increased odds ratio of depression for the lowest vs. highest vitamin D categories in the cross-sectional studies and a significantly increased hazard ratio of depression for the lowest vs. highest vitamin D categories in cohort studies (Anglin et al., 2013), raising the possibility that vitamin D supplementation may mitigate the development of depression symptoms. Other nutrients, such as B vitamins, omega-3 polyunsaturated fatty acids, zinc, and antioxidants, are also essential for neural functions. Reports show that the deficiency of these nutrients may result in altered memory function, cognitive impairment, and the development of a major depressive disorder (Sarris et al., 2015; Mikkelsen et al., 2016).

In addition to single macro- or micro-nutrient status, diet, dietary pattern, and eating behaviors were also altered among patients with depression symptoms. Converging evidence from laboratory, population research, and clinical trials suggest that a healthy diet and dietary patterns, such as the traditional Mediterranean-style diet and the Dietary Approach to Stop Hypertension diet, may lower the risk for depression (Opie et al., 2017, 2018; Khayyatzadeh et al., 2018). However, most reviews considered depression, in general, and a few imposed a focus on SAD. In addition, previous reviews on certain nutrients and their link to SAD concentrated on a single nutrient or class of nutrients, such as vitamin D.

Considering that there is limited research showing a broad picture of how eating style and nutrient intake changes in SAD patients, our current work aims to systematically review the evidence on whether diet and eating behavior are altered among the SAD population and how nutrition intervention influences the development of SAD.

## Methods

### Literature Search

**Figure 1:**
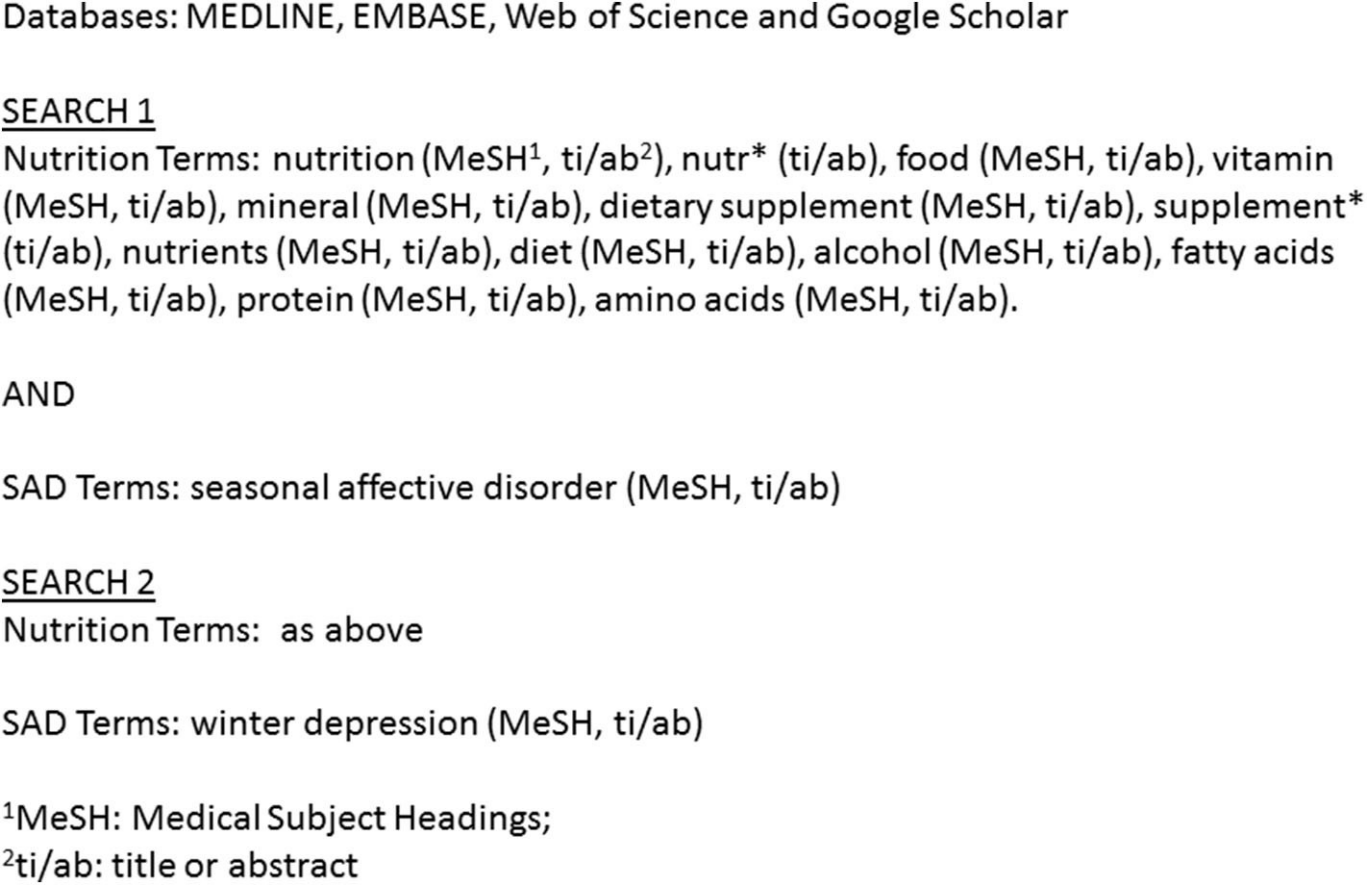
Search strategy.

We followed the Preferred Reporting Items for Systematic Reviews and Meta-Analyses guidelines for conducting a systematic review. We registered our search protocol on PROSPERO on July 4, 2019. We performed a comprehensive search of MEDLINE, EMBASE, Web of Science, and Google Scholar from inception up to July 1, 2019. A combination of Medical Subject Heading terms and keywords were input into each database. After all of the records were organized and duplicates were excluded, two independent investigators (Y. Yang, S. Zhang) screened the titles and abstracts. The search terms are listed in Figure 1.

### Eligibility

To obtain original studies on the effect of diet, eating behavior, and dietary supplements in SAD, we applied the following eligibility criteria and included studies if they have:

- patients with SAD confirmed by DSM-III-R, DSM-IV, DSM-IV-TR, and DSM-5, Rosenthal criteria for trials developed before DSM-III-R or patients with SAD screened by SPAQ
- included one of the following study designs: prospective cohort, cross-sectional, case-control (nested case-control and case-cohort), randomized controlled trials (RCTs), and baseline of intervention studies
- diet, dietary pattern, and eating behaviors measured with valid and established questionnaires
- outcomes related to SAD severity, mood, or changes in dietary or eating behavior and nutritional biomarkers
- provided a means or frequency report and statistical and/or epidemiological association measures

We excluded studies if the participants had other major depressive symptoms (such as bipolar disorder) or other types of physiological stress. We also excluded studies that assessed outcomes only by medication use, exemplified by noting the use of an antidepressant itself as the diagnosis of depression without employing a symptom questionnaire, screening measure, or diagnostic assessment. In addition, studies that gave nutrition supplementation through means other than oral administration were also excluded.

### Data Collection

Data collection was performed by two independent investigators (Y. Yang, S. Zhang) using a structured form. Minor discrepancies were resolved by consensus between the investigators. When major discrepancies occurred, a third investigator (J. Cheng) would be involved to make a final decision. When dealing with multiple endpoints, we only selected the outcomes that were related to SAD severity, mood changes, or the changes in diet or eating behavior and nutritional biomarkers. The following information was extracted from each included study: study characteristics (first author, study country, year of the study, study design, and sample size), participant characteristics (age), assessment of, or screening for, SAD (SAD screen tool), characteristics of exposure (diet or eating behavior, supplement type, and exposure assessment tool), outcome characteristics (outcome type, assessment tool), results (means or frequencies, comparison of the groups, and odds ratio), and conclusion. Extra information was extracted for RCTs: study duration, interventional group participant number, and control group participant number.

Tables were constructed to summarize the data. For studies showing statistical and epidemiological associations, the more complete measure of variability was presented (95% confidence intervals or *p-*value). The methods used for statistical analysis were listed with the conclusion of each study.

### Risk of Bias Assessment

Two independent investigators assessed the risk of bias in the included studies (X. Zhang, Y. Xu). Any disagreements were discussed between the investigators until a consensus was reached. The methodological quality of the included studies was evaluated by utilizing the Quality in Prognostic Studies tool for observational studies (Hayden et al., 2013) and the Cochrane Collaboration's tool for assessing the risk of bias in RCTs (Higgins et al., 2011).

The Quality in Prognostic Studies tool by Hayden et al. provided six important risk domains to consider when evaluating validity and bias in studies of prognostic factors: participation, attrition, prognostic factor measurement, confounding measurement and account, outcome measurement, and analysis and reporting (Hayden et al., 2013). Even though the Quality in Prognostic Studies tool was established for prognostic studies, “prognoses” are similar to “risks” in epidemiology (Sparling et al., 2017). The Cochrane Collaboration's tool covers six domains of bias: selection bias, performance bias, detection bias, attrition bias, reporting bias, and other bias. Within each domain, evaluations are made for one or more items to cover various aspects of the domain or different outcomes (Higgins et al., 2011).

The confounding domain was assessed based on potential confounders being controlled for and the method that was utilized to control them. Bennett et al. reported several socioeconomic and psychosocial variables that showed associations with both depression and eating behaviors, such as a history of depression, overall wellness status, social support, and income and employment, for example (Bennett et al., 2004). Studies that did not control for any of these confounders were rated as having a high risk of bias. Studies partially including these confounders were rated as having a medium risk of bias, and studies that accounted for all the confounders were rated as having a low risk of bias. In general, we rated the risk of bias by evaluating the prompting items within each domain. The judgments about the risk of bias for each domain were established based on the information on the prompting items provided by the studies, and each domain was rated as having a high, moderate, or low risk of bias after a comprehensive evaluation of the prompting items.

## Results

### Study Selection

**Figure 2:**
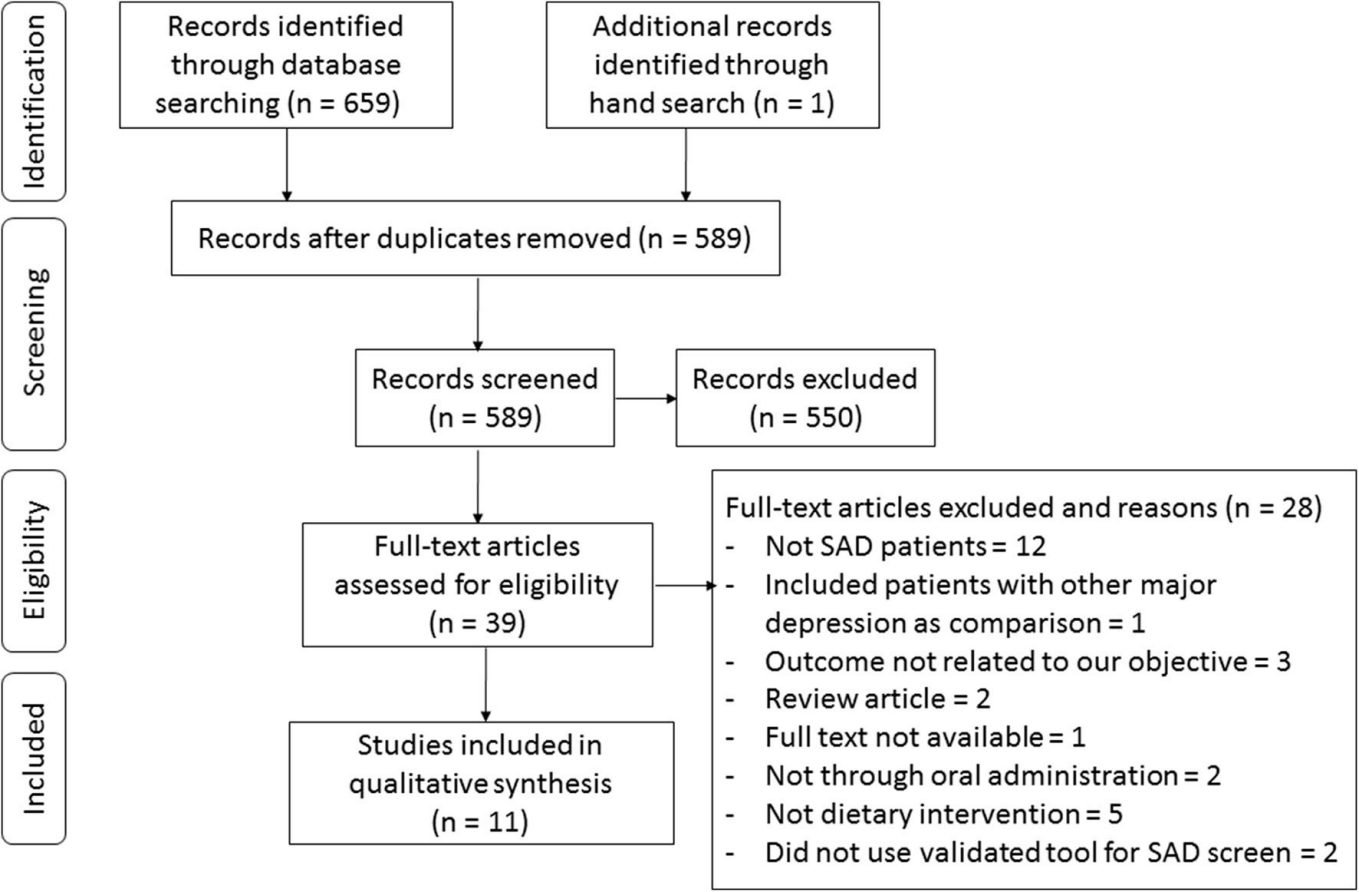
Flowchart of study selection according to the PRISMA guideline.

The process of study selection is presented in the flowchart in Figure 2. The search through the three electronic databases yielded 659 results, of which 589 were screened by title and abstract after excluding duplicates. Overall, 551 articles were excluded because they did not meet the inclusion criteria, which led to a total of 38 potential studies selected for full-text review. Among them, studies were excluded for not focusing on SAD (*n* = 12), including subjects with other major depression as comparisons (*n* = 1), outcomes unrelated to our objective (*n* = 3), being review articles (*n* = 2), the full text not being available (*n* = 1), supplementation being given not through oral administration (*n* = 2), not involving dietary intervention (*n* = 5), and not using a validated tool for SAD screening (*n* = 2). In total, we included data from 11 studies in the current systematic review (Krauchi and Wirz-Justice, 1988; Rosenthal et al., 1989; Berman et al., 1993; Oren et al., 1994; Krauchi et al., 1997; Danilenko et al., 2008; Mischoulon et al., 2010; Donofry et al., 2014; Frandsen et al., 2014; Meesters et al., 2016; Morales-Munoz et al., 2017).

Studies were grouped according to three categories of exposures: (1) diet or dietary patterns (*n* = 3), (2) eating behaviors (*n* = 4), and (3) nutrition supplementation (*n* = 5). Among them, one study fits two categories, so the information within this study was extracted separately. Specifically, the studies in categories 1 and 2 are observational studies with a total of 13,360 subjects, and the studies in category 3 are RCTs with 203 participants in total. The characteristics of the included studies evaluating the associations between diet, eating behaviors, and nutrition intervention and SAD are listed in Tables 1–3, respectively.

**Table 1:** Characteristics of included studies evaluating the associations between diet and SAD.

**First author, year, country**	**Study design, SAD screen tool**	**Total subjects, age (mean), Patients N**	**Diet, diet assessment tool**	**Outcome, outcome assessment tool**	**Means/** **frequencies**	**Comparison**	**OR (CI) or *p* values**	**Conclusion**
Meesters ANR, 2017, Finland (Study I)	Cross-sectional, SPAQ	4578, 52.1^a^, 123	Vegan, FFQ	SAD severity, SPAQ	14.6% among vegetarians, and 3.4% among non-vegetarians	Vegetarian vs. non-Vegetarian	**OR** **=** **3.9 (1.81–8.36)**	Being vegetarians is associated with higher possibility of developing SAD. However, logistic regression analysis revealed that none of the GSS item scores or the total GSS showed a significant relationship with vegetarianism.
				GSS, SPAQ	6.2 ± 3.6 for Vegetarians, and 5.2 ± 3.2 for non-Vegetarians	Vegetarian vs. non-Vegetarian	*p* > 0.05	
The Netherlands (Study II)	Cross-sectional, DSM-IV	257, 37.5, 257	Vegan, Asked by researchers whether being vegetarian	GSS, SPAQ	12.7 ± 4.4 for Vegetarians, and 13.5 ± 3.5 for non-Vegetarians	SAD vs. non-SAD	**OR** **=** **1.5** ***p*** **<** **0.05**	The logistic regression analysis showed a significant relationship between seasonal loss of energy and vegetarianism.
Morales-Muñoz I, 2017, Finland	Cross-sectional, SPAQ	8135, 55.7, 171	Alcoholism, M-CIDI	ADD, M-CIDI	0.08% for SAD patients, and 0.03% for control	Case vs. control	***p*** **<** **0.001**	The one-way ANOVA analysis showed that compared with control, people with SAD showed greater possibilities in having alcohol use/dependence disorder in lifetime.
				AUD, M-CIDI	0.12% for SAD patients, and 0.06% for control	Case vs. control	***p*** **=** **0.003**	
				Alcohol abuse during the past 12 months, M-CIDI	1.09% for SAD patients, and 1.02% for control	Case vs. control	***p*** **=** **0.002**	
				ADD during the past 12 months, M-CIDI	0.03% for SAD patients, and 0.01% for control	Case vs. control	***p*** **=** **0.032**	
				AUD during the past 12, M-CIDI	0.04% for SAD patients, and 0.01% for control	Case vs. control	***p*** **=** **0.024**	
				Seasonal changes in sleep duration, SPAQ	NR	One unit change in ADD	***p*** **=** **0.005**	Pearson correlation analysis showed that people diagnosed with ADD reported larger seasonal changes in sleep duration, social activity, energy, and mood; AUD patients reported larger seasonal changes in social activity and mood; patients suffered ADD past 12 months reported larger seasonal changes in sleep duration, social activity, energy, and mood; patients suffered AUD past 12 months reported larger seasonal changes in sleep duration, energy, and mood; and patients suffered alcohol abuse past 12 months reported larger seasonal changes in energy and mood.
				Seasonal change in social activity, SPAQ	NR	One unit change in ADD	***p*** **=** **0.007**	
				Seasonal changes in energy, SPAQ	NR	One unit change in ADD	***p*** **<** **0.001**	
				Seasonal changes in mood, SPAQ	NR	One unit change in ADD	***p*** **<** **0.001**	
				Seasonal changes in social activity, SPAQ	NR	One unit change in AUD	***p*** **=** **0.007**	
				Seasonal changes in mood, SPAQ	NR	One unit change in AUD	***p*** **<** **0.001**	
				Seasonal changes in sleep duration, SPAQ	NR	One unit change in ADD past 12 months	***p*** **=** **0.004**	
				Seasonal change in social activity, SPAQ	NR	One unit change in ADD past 12 months	***p*** **=** **0.003**	
				Seasonal changes in energy, SPAQ	NR	One unit change in ADD past 12 months	***p*** **=** **0.013**	
				Seasonal changes in mood, SPAQ	NR	One unit change in ADD past 12 months	***p*** **<** **0.001**	
				Seasonal changes in sleep duration	NR	One unit change in AUD past 12 months	***p*** **=** **0.004**	
				Seasonal changes in energy	NR	One unit change in AUD past 12 months	***p*** **=** **0.006**	
				Seasonal changes in mood	NR	One unit change in AUD past 12 months	***p*** **<** **0.001**	
				Seasonal changes in energy	NR	One unit change in alcohol abuse past 12 months	***p*** **=** **0.027**	
				Seasonal changes in mood	NR	One unit change in alcohol abuse past 12 months	***p*** **<** **0.001**	
Krauchi K, 1987, Switzerland	Case-control, DSM-III	54, 40.7^a^, 28	Food frequency, FDFQ	Starch-rich foods intake amount (portions/month), FDFQ	50.3 ± 15.1 for case, and 61.7 ± 15.5 for control	Case vs. Control	***p*** **<** **0.05**	One-way ANOVA showed that compare with control, SAD patients had more preference of consuming starch and fiber-rich foods, but similar frequencies of taking sugar-rich foods, dairy-rich foods, protein-rich foods, and caffeine-containing beverages.
				Fiber-rich foods intake amount (portions/month), FDFQ	61.6 ± 20.4 for case, and 82.8 ± 27.9 for control	Case vs. Control	***p*** **<** **0.01**	
				Sugar-rich foods intake amount (portions/month), FDFQ	34.4 ± 20.3 for case, and 41.3 ± 15.7 for control	Case vs. Control	*p* > 0.05	
				Protein-rich foods intake amount (portions/month)	26.4 ± 12.7 for case, and 26.2 ± 9.8 for control	Case vs. Control	*p* > 0.05	
				Dairy intake amount (portions/month)	59.4 ± 29.1 for case, and 53.5 ± 27.1 for control	Case vs. Control	*p* > 0.05	
				alcohol intake amount (dl/month)	5.0 ± 3.7 for case, and 4.4 ± 4.7 for control	Case vs. Control	*p* > 0.05	
				Caffeine-containing drinks amount (portions/month)	70.7 ± 14.7 for case, and 80.2 ± 21.7 for control	Case vs. Control	*p* > 0.05	
				Seasonal variation of starch-rich foods intake, FDFQ	NA	(Case vs. Control) * Seasons	*p* > 0.05	Two-way ANOVA for repeated measures analysis showed that there was no interaction between case vs. control and seasonality on seasonal variation of food item intake. However, significant seasonal variations were found in starch-rich foods and dairy products in cases.
				Seasonal variation of fiber-rich foods intake amount, FDFQ	NA	(Case vs. Control) * Seasons	*p* > 0.05	
				Seasonal variation of sugar-rich foods intake amount, FDFQ	NA	(Case vs. Control) * Seasons	*p* > 0.05	
				Seasonal variation of protein-rich foods intake amount, FDFQ	NA	(Case vs. Control) * Seasons	*p* > 0.05	
				Seasonal variation of dairy intake amount, FDFQ	NA	(Case vs. Control) * Seasons	*p* > 0.05	
				Seasonal variation of alcohol intake amount, FDFQ	NA	(Case vs. Control) * Seasons	*p* > 0.05	
				Seasonal variation of caffeine-containing drinks amount, FDFQ	NA	(Case vs. Control) * Seasons	*p* > 0.05	

FFQ, Food Frequency Questionnaire; SPAQ, Seasonal Pattern Assessment Questionnaire; GSS, Global Seasonality Score; DSM, Diagnostic and Statistical manual of Mental disorders; M-CIDI, The World Health Organization Composite International Diagnostic Interview, the Munich version; ADD, Alcohol dependence disorder in lifetime; AUD, Alcohol use disorder in lifetime; SIGH-SAD, Structured Interview Guide for the Hamilton Depression Rating Scale, Seasonal Affective Disorders; SCID, DSM-IV Structured Clinical Interview for Depression; NR, Not report; Underlined context, detailed data not shown in the publication; Bold text, Statistically significant.

a *Calculated according to the information provided*.

**Table 2:** Characteristics of included studies evaluating the associations between eating behaviors and SAD.

**First author, year, country**	**Study design, SAD screen tool**	**Total subjects, age (mean), Patients N**	**Diet, diet assessment tool**	**Outcome, outcome assessment tool**	**Means/** **frequencies**	**Comparison**	**OR (CI) or *p* values**	**Conclusion**
Donofry SD, 2014, USA	Cross-sectional, DSM-IV-TR	112, 41.8, 112	Binge eating, QEWP-R	Binge eating, QEWP-R	26.5% among SAD patients	NA	NA	Logistic regression adjusting for age and gender showed that the spectrum of eating pathology in SAD patients involve binge eating
				Weekly binge eating, QEWP-R	11.6% among SAD patients	NA	NA	
				Binge eating defined by DSM-IV-TR, QEWP-R	8.9% among SAD patients	NA	NA	
				GSS, SPAQ	16.3 ± 3.6 for binge eating patients, and 14.9 ± 4.2 for non-clinical subjects	Binge eating patients vs. non-clinical subjects	OR = 1.057, *p* = 0.40	
Krauchi K, 1996, Switzerland	Case-control, Rosenthal criteria or DSM-III-R	164, NR, 84	Eating disorders, DEBQ	External eating, DEBQ	NR	Case vs. Control	***p*** **<** **0.001**	One-way ANOVA with Bonferroni adjustment showed that compared with normal control and stressful subjects (control II), SAD patients had higher tendency for 'external eating' (food intake steered by external stimuli) and 'emotional eating' (eating simulated by anxiety, insecurity, irritability, and depression), but similar 'restraint eating' (restrict food intake for weight control).
				Emotional eating, DEBQ	NR	Case vs. Control	***p*** **<** **0.001**	
				Restraint eating, DEBQ	NR	Case vs. Control	*p* > 0.05	
			Conditional food intake, Added questions to DEBQ	Consume sweets when depressed, Added questions to DEBQ	57.1% for case, and 13.2% for control	Case vs. Control	***p*** **<** **0.05**	One-way ANOVA with Bonferroni adjustment showed that compared with normal control and stressful subjects (control II), the percentage of consuming sweets when experiencing emotional eating (depression, anxiety, loneliness) was higher among SAD patients. Conditional food intake of other food items were similar between case and control during external eating and restraint eating.
				Consume starch when depressed, Added questions to DEBQ	17.9% for case, and 2.6% for control	Case vs. Control	*p* > 0.05	
				Consume fruits when depressed, Added questions to DEBQ	2.4% for case, and 0% for control	Case vs. Control	*p* > 0.05	
				Consume caffeine when depressed, Added questions to DEBQ	21.4% for case, and 21.1% for control	Case vs. Control	*p* > 0.05	
				Consume alcohol when depressed, Added questions to DEBQ	14.3% for case, and 2.6% for control	Case vs. Control	*p* > 0.05	
				Consume dairy products when depressed, Added questions to DEBQ	11.9% for case, and 5.3% for control	Case vs. Control	*p* > 0.05	
				Consume sweets when anxious, Added questions to DEBQ	27.4% for case, and 5.3% for control	Case vs. Control	***p*** **<** **0.05**	
				Consume starch when anxious, Added questions to DEBQ	10.7% for case, and 0% for control	Case vs. Control	*p* > 0.05	
				Consume fruits when anxious, Added questions to DEBQ	3.6% for case, and 0% for control	Case vs. Control	*p* > 0.05	
				Consume caffeine when anxious, Added questions to DEBQ	8.3% for case, and 10.5% for control	Case vs. Control	*p* > 0.05	
				Consume alcohol when anxious, Added questions to DEBQ	16.7% for case, and 10.5% for control	Case vs. Control	*p* > 0.05	
				Consume dairy products when anxious, Added questions to DEBQ	9.5% for case, and 2.6% for control	Case vs. Control	*p* > 0.05	
				Consume sweets when lonely, Added questions to DEBQ	38.1% for case, and 7.9% for control	Case vs. Control	***p*** **<** **0.05**	
				Consume starch when lonely, Added questions to DEBQ	9.5% for case, and 5.3% for control	Case vs. Control	*p* > 0.05	
				Consume fruits when lonely, Added questions to DEBQ	4.8% for case, and 2.6% for control	Case vs. Control	*p* > 0.05	
				Consume caffeine when lonely, Added questions to DEBQ	10.7% for case, and 7.9% for control	Case vs. Control	*p* > 0.05	
				Consume alcohol when lonely, Added questions to DEBQ	14.3% for case, and 2.6% for control	Case vs. Control	*p* > 0.05	
				Consume dairy products when lonely, Added questions to DEBQ	7.1% for case, and 2.6% for control	Case vs. Control	*p* > 0.05	
				Consume sweets when bored, Added questions to DEBQ	20.2% for case, and 15.8% for control	Case vs. Control	*p* > 0.05	
				Consume starch when bored, Added questions to DEBQ	9.5% for case, and 7.9% for control	Case vs. Control	*p* > 0.05	
				Consume fruits when bored, Added questions to DEBQ	7.1% for case, and 10.5% for control	Case vs. Control	*p* > 0.05	
				Consume caffeine when bored, Added questions to DEBQ	10.7% for case, and 13.2% for control	Case vs. Control	*p* > 0.05	
				Consume alcohol when bored, Added questions to DEBQ	6.0% for case, and 2.6% for control	Case vs. Control	*p* > 0.05	
				Consume dairy products when bored, Added questions to DEBQ	8.3% for case, and 2.6% for control	Case vs. Control	*p* > 0.05	
				Consume sweets during external eating, Added questions to DEBQ	22.6% for case, and 15.8% for control	Case vs. Control	*p* > 0.05	
				Consume starch during external eating, Added questions to DEBQ	6.0% for case, and 2.6% for control	Case vs. Control	*p* > 0.05	
				Consume crackers during external eating, Added questions to DEBQ	2.4% for case, and 0% for control	Case vs. Control	*p* > 0.05	
				Consume protein during external eating, Added questions to DEBQ	1.2% for case, and 2.6% for control	Case vs. Control	*p* > 0.05	
				Consume fruits during external eating	1.2% for case, and 2.6% for control	Case vs. Control	*p* > 0.05	
				Consume caffeine during external eating, Added questions to DEBQ	6.0% for case, and 13.2% for control	Case vs. Control	*p* > 0.05	
				Consume alcohol during external eating, Added questions to DEBQ	14.3% for case, and 10.5% for control	Case vs. Control	*p* > 0.05	
				Consume dairy products during external eating, Added questions to DEBQ	2.4% for case, and 2.6% for control	Case vs. Control	*p* > 0.05	
				Consume sweets during restraint eating, Added questions to DEBQ	53.6% for case, and 42.1% for control	Case vs. Control	*p* > 0.05	
				Consume starch during restraint eating, Added questions to DEBQ	33.3% for case, and 15.8% for control	Case vs. Control	*p* > 0.05	
				Consume protein during restraint eating, Added questions to DEBQ	7.1% for case, and 10.5% for control	Case vs. Control	*p* > 0.05	
				Consume fat during restraint eating, Added questions to DEBQ	19.0% for case, and 23.7% for control	Case vs. Control	*p* > 0.05	
				Consume alcohol during restraint eating, Added questions to DEBQ	6.0% for case, and 10.5% for control	Case vs. Control	*p* > 0.05	
Berman K, 1993, Canada	Case-control, DSM-III-R	60, 32.8^a^, 30	Dysfunctional eating, EDI	Bulimia, EDI	3.3 ± 4.2 for case, and 0.9 ± 1.9 for control	Case vs. Control	***p*** **<** **0.05**	*post hoc* student-newman-keuls tests for pairwise comparisons (case vs. control vs. bulimia nervosa patients) showed more severe bulimia disorders in SAD patients compared to non-clinical subjects.
Krauchi K, 1987, Switzerland	Case-control, DSM-III	54, 40.7^a^, 28	Food frequency, FDFQ	Breakfast in weekdays (meal/month), FDFQ	21.0 ± 5.1 for case, and 15.4 ± 9.6 for control	Case vs. Control	***p*** **<** **0.05**	One-way ANOVA showed that compare with control, SAD patients consumed significantly more dinners and evening snacks during weekdays and weekends.
				Morning snacks in weekdays (meal/month), FDFQ	6.1 ± 6.5 for case, and 6.7 ± 7.2 for control	Case vs. Control	*p* > 0.05	
				Lunch in weekdays (meal/month), FDFQ	20.5 ± 6.2 for case, and 21.1 ± 3.6 for control	Case vs. Control	*p* > 0.05	
				Afternoon snacks in weekdays (meal/month), FDFQ	8.7 ± 7.0 for case, and 6.1 ± 6.6 for control	Case vs. Control	*p* > 0.05	
				Dinner in weekdays (meal/month), FDFQ	22.7 ± 3.7 for case, and 21.1 ± 4.0 for control	Case vs. Control	***p*** **<** **0.05**	
				Evening snacks in weekdays (meal/month), FDFQ	8.6 ± 7.5 for case, and 2.9 ± 3.7 for control	Case vs. Control	***p*** **<** **0.001**	
				Breakfast in weekends (meal/month), FDFQ	2.1 ± 1.6 for case, and 1.8 ± 1.5 for control	Case vs. Control	*p* > 0.05	
				Morning snacks in weekends (meal/month), FDFQ	0.1 ± 0.4 for case, and 0.2 ± 0.5 for control	Case vs. Control	*p* > 0.05	
				Brunch in weekends (meal/month), FDFQ	2.0 ± 1.5 for case, and 1.7 ± 1.4 for control	Case vs. Control	*p* > 0.05	
				Lunch in weekends (meal/month), FDFQ	1.4 ± 1.5 for case, and 1.6 ± 1.4 for control	Case vs. Control	*p* > 0.05	
				Afternoon snacks in weekends (meal/month), FDFQ	1.7 ± 1.2 for case, and 1.2 ± 1.0 for control	Case vs. Control	*p* > 0.05	
				Dinner in weekends (meal/month), FDFQ	3.8 ± 0.5 for case, and 3.4 ± 0.7 for control	Case vs. Control	***p*** **<** **0.01**	
				Evening snacks in weekends (meal/month), FDFQ	1.3 ± 1.4 for case, and 0.5 ± 0.5 for control	Case vs. Control	***p*** **<** **0.05**	

DSM, Diagnostic and Statistical manual of Mental disorders; QEWP-R, Questionnaire on Eating and Weight Patterns-Revised; DEBQ, Dutch Eating Behavior Questionnaire; SPAQ, Seasonal Pattern Assessment Questionnaire; GSS, Global Seasonality Score; SCID, DSM-IV Structured Clinical Interview for Depression; FDFQ, Food/Drink Frequency Questionnaire; EDI, Eating Disorders Inventory; NA, Not available; NR, Not report; Bold text, Statistically significant.

a *Calculated according to the information provided*.

**Table 3:** Characteristics of included studies evaluating the associations between nutrition intervention and SAD.

**First author, year, country**	**Study design, SAD screen tool**	**Total N, age (mean)**	**Interv, dosage/d, duration**	**Ctrl**	**Interv N**	**Ctrl N**	**Outcome, outcome assessment tool**	**(Mean ± SD)/frequency in interv group**	**(Mean ± SD)/frequencies in ctrl group**	**Compare, *p*-values**	**Conclusion**
Frandsen TB, 2014, Denmark	RCT, SPAQ	43, 44.2	Vitamin D, 70 μg, 3 months	Placebo	22	21	Depression severity, SIGH-SAD	−6.4 ± 3.3^*^	−6.8 ± 9.5^*^	Interv vs. ctrl, *p* = 0.7	One-way ANOVA analysis showed that compared with the control group, vitamin D supplementation presented no effect in impeding SAD.
Mischoulon D, 2010, USA	RCT, crossover, SCID	18, 43	High carb^a^ for phase I and ctrl^b^ for phase II, Twice, 12 days for each phase	Ctrl^b^ for phase I and high carb^a^ for phase II	10	8	Depression severity, Hamilton-D-28 scale	6.5 ± 3.8 at the end of phase I, and 4.6 ± 3.9 at the end of phase II	9.3 ± 4.4 at the end of phase I, and 7.9 ± 5.3 at the end of phase II	Interv vs. ctrl, *p* = 0.09	Repeated measure ANOVA showed that the high-carbohydrate mix group did not significantly decrease depression symptom or remission compared with the control group.
							Remission rates, Hamilton-D-28 scale	50% at the end of phase I, and 88% at the end of phase II	38% at the end of phase I, and 50% at the end of phase II	Interv vs. ctrl, *p* = 0.66 at the end of phase I, and *p* = 0.28 at the end of phase II	
	RCT, SCID	32, 46	High carb^c^, Twice, 21 days	ctrl^b^	15	17	Depression severity, Hamilton-D-28 scale	6.4 ± 5.8	6.1 ± 4.4	Interv vs. ctrl, *p* = 0.88	Repeated measure ANOVA showed that the high-carbohydrate mix group did not significantly decrease depression symptom or remission compared with the control group.
							Remission rates, Hamilton-D-28 scale	71%	71%	Interv vs. ctrl, *p* = 1	
Danilenko KV, 2008, Russia	RCT, DSM-IV	22, 37.8	High carb (morning), NA, 9 days	NA	9	NA	Depression severity, SIGH-SAD	NR	NR	[High carb (morning) vs. high carb (evening) vs. high protein] × time, *p* = 0.61	Repeated measure ANOVA showed that no differential effects of diet on depression were found between high carb diet vs. high protein diet. However, participants from all groups had an improved SIGH-SAD score.
			High carb (evening), NA, 9 days	NA	6	NA					
			High protein, NA, 9 days	NA	7	NA					
							Emotional eating, DEBQ	NR	NR	[High carb (morning) vs. high carb (evening) vs. high protein] × time, ***p*** **=** **0.014**	Repeated measure ANOVA revealed that subjects' ratings of the 'emotional' factor increased after the CHO-morning diet, decreased after the CHO-evening diet, and remained unchanged after the high-protein diet. Diet significantly affected subjects' eating behavior, and such impact was affected by time. Ratings of restrained and external eating factors were similar before and after the diets, and there was no significant difference among meal groups.
							External eating, DEBQ	NR	NR	[High carb (morning) vs. high carb (evening) vs. high protein] × time, p > 0.05	
							Restraint eating, DEBQ	NR	NR	[High carb (morning) vs. high carb (evening) vs. high protein] × time, *p* > 0.05	
Oren DA, 1994, USA	RCT, DSM-III-R	27, 48	Vitamin B12, 4.5 mg, 2 weeks	Placebo	14	13	Depression severity, SIGH-SAD	18 ± 8	21 ± 10	Interv vs. ctrl, *p* > 0.25	Repeated measure ANOVCA showed that cyanocobalamin supplementation did not change SIGH-SAD rating scores between interventional group and control group.
Rosenthal NE, 1988, USA	RCT, crossover, DSM-III	32 (16 SAD patients and 16 non-clinical subjects), 40	High carb diet^b^ follows High protein diet (sequence 1), NA, one-time intervention	High protein diet^c^ follows High carb diet (sequence 2)	16	16	Tension, POMS	NR	NR	Meal (carb vs. prot) x sequence, ***p*** **<** **0.04**	Repeated measure ANOVA showed that the high-carb meal significantly decreased tension, depression, and anger scores, whereas the protein-rich meal had the opposite effect. The effect of the meals was affected by sequence. Fatigue values increased following the protein-rich meal in both patients and controls. High carb diet decreased fatigue in patients but not in control. Vigor declined in the first 2 h after both meals, but the decline was less marked in the SAD group than in normal controls after high carb diet.
							Depression, POMS	NR	NR	Meal x sequence, ***p*** **<** **0.02**	
							Anger, POMS	NR	NR	Meal x sequence, ***p*** **<** **0.05**	
							Fatigue, POMS	NR	NR	Meal x group (SAD vs. ctrl) x time, ***p*** **<** **0.02**	
							Vigor, POMS	NR	NR	Diet x sequence, ***p*** **<** **0.05**	

Interv, intervention; Ctrl, control; FFQ, Food Frequency Questionnaire; SPAQ, Seasonal Pattern Assessment Questionnaire; GSS, Global Seasonality Score; DSM, Diagnostic and Statistical manual of Mental disorders; M-CIDI, The World Health Organization Composite International Diagnostic Interview, the Munich version; ADD, Alcohol dependence disorder in lifetime; AUD, Alcohol use disorder in lifetime; SIGH-SAD, Structured Interview Guide for the Hamilton Depression Rating Scale, Seasonal Affective Disorders; SCID, DSM-IV Structured Clinical Interview for Depression; NR, Not report; Underscored context, detailed data not shown; Bold text, Statistically significant.

* Mean of difference ± SD.

a 40 grams of a mixture of potato starch, maltodextrin, dextrose, and dextrin.

b Carbohydrate-protein mix consisting of 15 g milk protein casein and 25 g carbohydrate mixture.

c Contains 105 g of carbohydrate, 0.7 g of protein, and 42.7 g of fat.

d *Contains 105 g of protein, 15 g of carbohydrates, and 33.3 of fat*.

### Diet, Dietary Pattern, and SAD

#### Vegetarianism

One cross-sectional study investigated the possible link between vegetarianism and SAD (Meesters et al., 2016). By using available data from the Finnish national FINRISK 2012 study and data from the outpatient clinic for SAD in the Netherlands, the author of that study found an association between being vegetarian and SAD. They revealed that 14.6% of the population that claimed themselves as “vegetarians” suffered from SAD, while among the non-vegetarian participants, only 3.4% of them were SAD patients. The proportion of vegetarians in the SAD patients from the Dutch SAD outpatient clinic dataset was 12.5%, which was higher than that in the general population (4.5%). However, the association between being vegetarian and experiencing a loss of energy was not consistent in Finland vs. the Netherlands.

#### Alcoholism

Two studies assessed alcohol drinking among SAD patients vs. the control population (Krauchi and Wirz-Justice, 1988; Morales-Munoz et al., 2017). One population-based, cross-sectional study showed that, compared with the control, people with SAD showed greater possibilities of having alcohol dependence in their lifetime, represented by a higher prevalence of alcohol dependence disorder in their lifetime, alcohol use disorder (AUD) in their lifetime, alcohol abuse during the past 12 months, alcohol dependence disorder during the past 12 months, or AUD during the past 12 months (Morales-Munoz et al., 2017). It is important to note that the DSM-5 changed from differentiating between alcohol abuse and alcohol dependence to a single category of AUD, which is defined as alcohol often being taken in larger amounts or over a longer period than intended. By further exploring the relationship between alcoholism and Global Seasonality Score items, the researchers found that people with comorbid alcohol abuse showed larger seasonal changes in sleep duration, social activity, energy, and mood, which implicated a greater severity of seasonal complaints. No significant association between alcohol abuse and seasonal weight or appetite changes was found in this study. On the contrary, one case-control reported comparable alcohol consumption amounts between the SAD patients and the control subjects, and seasonality variation was not observed in alcohol consumption among SAD patients (Krauchi and Wirz-Justice, 1988).

#### Food Intake Frequency

Food intake frequency in SAD was reported by one case-control study (Krauchi and Wirz-Justice, 1988). Krauchi et al. reported that, compared to the control, SAD patients have more preference for consuming starch and fiber-rich foods but similar frequencies of taking sugar-rich foods, dairy-rich foods, protein-rich foods, and caffeine-containing beverages. Furthermore, the researchers found that, in comparison to controls, SAD subjects showed a significantly increased starch-rich food score in all seasons except summer (*p* < 0.05) and a minimal dairy product intake in winter, compared to all other seasons (*p* < 0.01). Exploring the seasonal variation of food consumption further showed that there was no interaction between case vs. control and seasonality on seasonal variation of food item intake. However, significant seasonal variations were found in starch-rich foods and dairy products in SAD patients.

### Eating Behavior and SAD

#### Binge Eating and Restraint Eating

One cross-sectional study examined the frequency of binge eating among SAD patients using a food and drink frequency questionnaire (Donofry et al., 2014). This study showed that 26.5% of the subjects with SAD self-reported episodic overeating with a loss of control, 11.6% had weekly binge eating, and 8.9% had binge eating defined by the DSM-IV-TR. “Restraint” eating is based on the tendency to restrict food consumption to maintain body weight or to promote weight loss, such as with anorexia nervosa (Wardle, 1987). One case-control study reported that the difference in the restraint eating factor scores between SAD patients and control was insignificant (Krauchi et al., 1997). By leveraging the Eating Disorders Inventory, one case-control study revealed significantly more severe bulimia nervosa, which is an eating disorder characterized by a cycle of binging and compensatory behaviors exemplified by self-induced vomiting (Castillo and Weiselberg, 2017) in SAD patients compared to non-clinical subjects (Berman et al., 1993).

#### External Eating and Emotional Eating

External eating refers to whether persons eat more than normal under the situations of external stimuli: the sight, smell, amount, and availability of food; the time-of-day signal or the lack of clearly recognized internal signals of hunger and satiation (Nisbett, 1968; Schachter and Gross, 1968; Nisbett and Kanouse, 1969). Emotional eating manifests itself through increased eating under the situations or emotions of anxiety, insecurity, irritability, or depression (Bruch, 1961). To investigate the eating style of SAD patients, Krauchi et al. conducted a case-control study and found that SAD patients had higher frequencies of external eating and emotional eating (Krauchi et al., 1997). In addition, the researchers reported that, when encountering depression, anxiety, and loneliness, SAD subjects had a higher tendency to consume sweets than normal controls. However, the frequency of consuming starch, fruits, caffeine, alcohol, and dairy was comparable between case and control under external and emotional situations.

#### Meal Frequency

One case-control study reported meal frequency within different time frames in either weekdays or weekends by recruiting 28 SAD patients and 26 controls (Krauchi and Wirz-Justice, 1988). Data showed that SAD patients consumed significantly larger dinners and more evening snacks during weekdays and weekends, while the frequencies of morning snack, lunch, and afternoon snack intake on both weekdays and weekends and brunch intake on weekends were similar between case and control.

### Nutrition Intervention and SAD

#### Vitamin D

One RCT reported the efficacy of vitamin D supplementation in SAD patients (Frandsen et al., 2014). The participants were randomized to either 70 μg of vitamin D or placebo for 12 weeks during the winter period. At the end of the intervention, there were no significant between-group differences in the sum of the self-reported questionnaire, the Structured Interview Guide for the Hamilton Depression Rating Scale, and Seasonal Affective Disorders (SIGH-SAD).

#### Vitamin B12

The treatment effect of vitamin B12 (cyanocobalamin) for SAD symptoms was reported in one RCT (Oren et al., 1994). After supplementing the interventional group with vitamin B12 for 2 weeks, the study did not observe any significant difference in SIGH-SAD rating scores between the interventional group and the control group.

#### Macronutrients

Three RCTs examined the impact of oral carbohydrate administration in individuals with SAD (Rosenthal et al., 1989; Danilenko et al., 2008; Mischoulon et al., 2010). One crossover RCT recruited both SAD patients and normal controls into the study and randomly provided all of the participants either a carbohydrate-rich diet followed by a protein-rich diet (sequence 1) or a protein-rich diet followed by a high-carb diet (sequence 2) (Rosenthal et al., 1989). Results showed that the high-carb meal significantly decreased tension, depression, and anger scores, whereas the protein-rich meal had the opposite effect. The effect of the meals on tension, depression, and anger was affected by sequence. Fatigue values increased following the protein-rich meal in both patients and controls. The high-carb diet decreased fatigue in patients but not in the control. Vigor declined in the first 2 h after both meals, but the decline was less marked in the SAD group than in normal controls after the high-carb diet. Such results were not in line with the findings from another two RCTs, which reported insignificant effects of high-carb diet administration on SAD severity or remission among subjects with SAD symptoms (Danilenko et al., 2008; Mischoulon et al., 2010).

In addition, Danilenko et al. separated the carbohydrate-rich diet into two different time frames, morning and evening, and compared the role of the high-carb (morning) and high-carb (evening) diets with a high-protein diet in SAD progression. Although the amelioration of SAD severity between diet groups was statistically insignificant, the researchers found distinctively altered eating behavior in different groups. Subjects' ratings of the “emotional” factor increased after the carb-morning diet, decreased after the carb-evening diet, and remained unchanged after the high-protein diet, and the impact of diet was affected by time. The emotional factor is constructed from a questionnaire with 13 questions expressing the desire to eat following negative emotions. Ratings of restrained and external eating factors were similar before and after the diets, and there was no significant difference among meal groups (Danilenko et al., 2008).

### Methodological Quality

**Figure 3:**
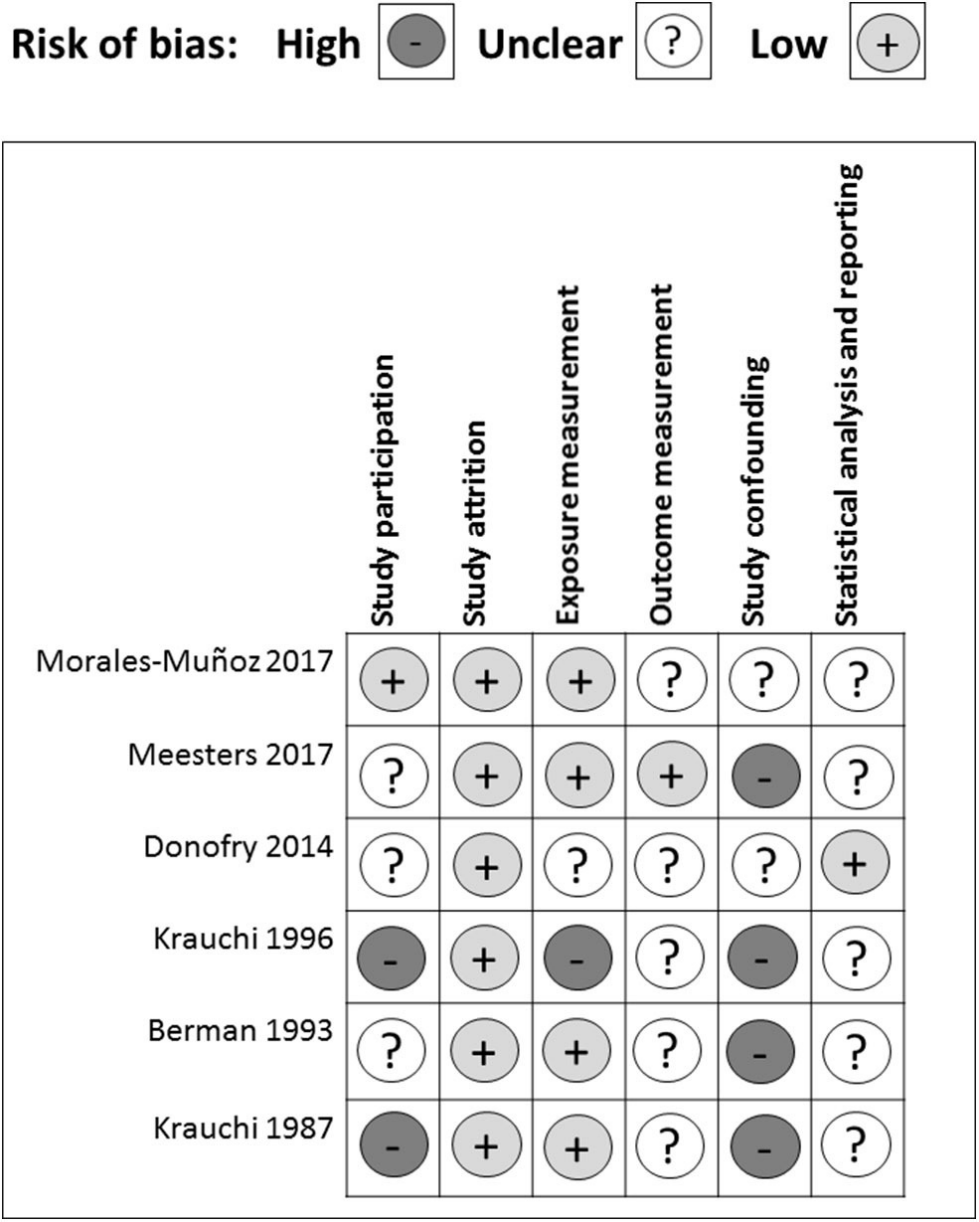
Risk of bias assessment for observational studies.

**Figure 4:**
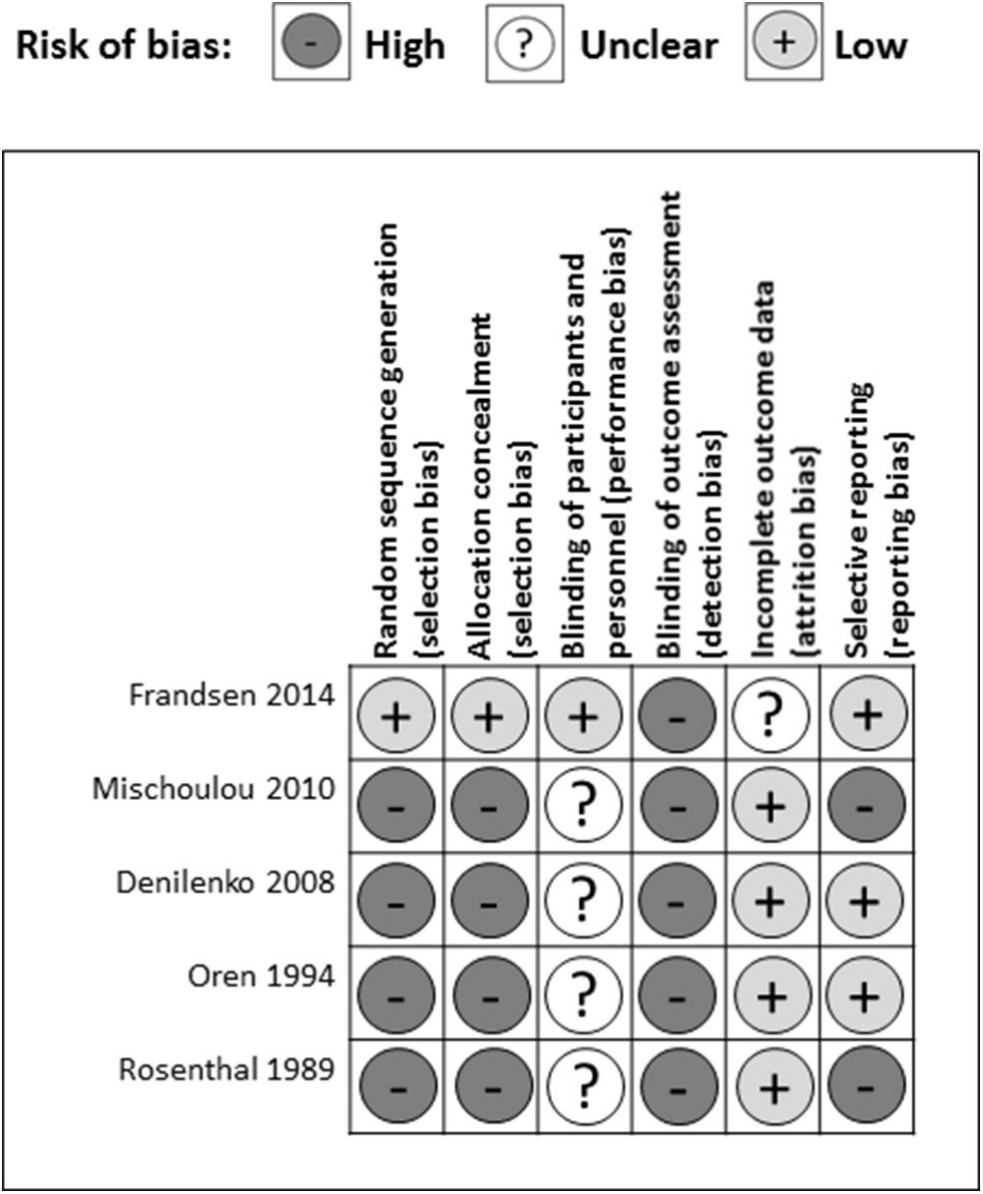
Risk of bias assessment for RCTs.

There were several common sources of bias in the included studies, which are presented in Figures 3, 4. Since the risk of bias in certain domains (such as selection bias) was different between observational studies and clinical trials, we utilized different tools for bias assessment, and thus, the bias of these studies was reported separately.

#### Observational Studies

All of the cross-sectional and case-control studies presented several risks of bias in one or more domains. In the study participation domain, only one study achieved a low risk of bias, while 50% (3/6) had a medium risk and 33% (2/6) had a high risk of bias (Figure 3). Specifically, three studies did not enroll adequate participants (Berman et al., 1993; Krauchi et al., 1997; Donofry et al., 2014). One study did not provide descriptions of the source population (Krauchi and Wirz-Justice, 1988). Two studies did not report a detailed period or place of recruitment (Krauchi et al., 1997; Donofry et al., 2014), and three studies did not provide clear inclusion and exclusion criteria (Krauchi and Wirz-Justice, 1988; Berman et al., 1993; Krauchi et al., 1997). Only one study provided an adequate description of the sampling frame and recruitment (Morales-Munoz et al., 2017), and all of the studies reported details of the baseline study sample. Overall, the observational studies included in this systematic review had a low bias in the study attrition domain since either no, or only short-period, follow-ups were required throughout the study.

Four out of six studies acquired a low risk of bias in the exposure measurement domain. One had a medium risk, and one had a high risk of bias. All of the studies but one (Krauchi et al., 1997) provided a clear definition of prognostic factor. Two studies did not employ valid prognostic factor measurement (Krauchi and Wirz-Justice, 1988; Donofry et al., 2014). Four studies only presented categorical prognostic factor data or did not articulate the cut-off points of their continuous variables (Krauchi and Wirz-Justice, 1988; Krauchi et al., 1997; Donofry et al., 2014; Meesters et al., 2016). Only one study stated that the method of prognostic factor measurement was not the same for all study participants, and the proportion of the study sample that completed data for prognostic factors was inadequate (Krauchi et al., 1997).

In the outcome measurement domain, only one study had a low risk of bias, while the other five studies presented a medium risk of bias, which resulted from an unclear definition of outcome (Krauchi and Wirz-Justice, 1988; Morales-Munoz et al., 2017), inadequately valid outcome measurement methods (Krauchi and Wirz-Justice, 1988; Kurlansik and Ibay, 2012; Donofry et al., 2014), and inconsistent outcome measurements for each study participant (Berman et al., 1993). Since most studies used analysis of variance and did not perform correlation analysis (Krauchi and Wirz-Justice, 1988; Berman et al., 1993; Krauchi et al., 1997; Meesters et al., 2016), they did not adjust for potential confounders. For the two studies that used logistic regression to explore the relationship between prognostic factors and SAD severity, Donofry et al. adjusted for age and gender (Donofry et al., 2014), while Meesters et al. adjusted for age and gender in the Dutch data, and only gender in the Finnish data (Meesters et al., 2016). Thus, in the study-confounding domain, two studies had a medium risk of bias, whereas all of the other studies had a high risk of bias.

In the statistical analysis and reporting domain, one study was evaluated as having a low risk of bias, while the others had a medium risk of bias, mostly resulting from an insufficient strategy of model planning (Krauchi and Wirz-Justice, 1988; Berman et al., 1993; Krauchi et al., 1997; Meesters et al., 2016), and an inadequate statistical model for the design of the study (Krauchi and Wirz-Justice, 1988; Berman et al., 1993; Krauchi et al., 1997; Donofry et al., 2014; Meesters et al., 2016; Morales-Munoz et al., 2017). One study was subjected to reporting bias due to the incomplete presentation of study results (Morales-Munoz et al., 2017). In addition, all of the cross-sectional and case-control studies used questionnaires retrospectively because of the nature and characteristics of observational studies and, may, therefore, have been influenced by recall bias.

#### RCTs

Out of five RCTs, only one study reported a detailed method of random sequence generation and allocation concealment (Frandsen et al., 2014) leaving four studies at high risk of selection bias (Rosenthal et al., 1989; Oren et al., 1994; Danilenko et al., 2008; Mischoulon et al., 2010) (Figure 4). One study articulated the process of blinding participants and personnel (Frandsen et al., 2014), while the others only stated that they had double-blinded the subjects (Rosenthal et al., 1989; Oren et al., 1994; Danilenko et al., 2008; Mischoulon et al., 2010) resulting in one study having a low risk and four studies a high risk of performance bias. None of the studies provided detailed descriptions of the blinding of outcome assessment, leading to a high risk of detection bias among five studies. In the attrition bias domain, four out of five studies had a low risk of bias, while one study had a medium risk of bias resulting from incomplete outcome data (Frandsen et al., 2014). In the selective reporting domain, three studies had a low risk of bias, while two studies were scored as having a high risk of bias with missing appetite or food craving data (Mischoulon et al., 2010), and missing between-group analysis for tension, depression, and anger (Rosenthal et al., 1989).

## Discussion

Overall, there is lacking evidence that diet, eating behavior, and nutrition intervention influence the development of SAD. The observational studies, including one for vegetarianism; two for alcoholism; one for food intake frequency; two for binge eating, restraint eating, external eating, and emotional eating; and one for meal frequency, revealed some distinctive dietary and behavioral patterns in SAD patients, however. Among the five RCTs, three studies reported inconsistent efficacy of carbohydrate-rich diets in improving SAD symptoms, and the two other studies found no evidence of an association between vitamin D supplementation or vitamin B12 supplementation and SAD. Nevertheless, given the methodological limitations of the studies and the inadequacy of publications on this topic, the lack of evidence does not necessarily imply the absence of true associations. We did not conduct a meta-analysis due to the heterogeneity of the studies. However, this review is the most current synthesis of the evidence revealing the role of diet, eating behavior, and nutrition intervention in SAD patients.

In the study by Meesters et al., the percentage of vegetarians that suffered from SAD in Finland was four times higher than the percentage of people with SAD symptoms in the control population. The percentage of vegetarian SAD patients in the Netherlands dataset was three times higher than that of vegetarians in the control population (Meesters et al., 2016). These findings were consistent with the report from three other observational research works showing that vegetarians had higher depression scores, on average, than non-vegetarians (Baines et al., 2007; Michalak et al., 2012; Hibbeln et al., 2018). Several possible factors previously linked to an augmented risk of depressive symptoms might underlie the increased risk of depression among vegetarians, including the inadequate intake of multiple interactive nutrients that might be lacking in vegetarian diets. L-tryptophan, for example, is abundant in animal-sourced protein and is a vital nutrient for serotonin synthesis in the brain (Lambert et al., 2002). Endogenous serotonin is one of the major monoamine transmitters implicated in mood disorders (Gupta et al., 2013). Any disruption in the synthesis, metabolism, or uptake of serotonin has been found to be partly responsible for certain manifestations of depression, such as fatigue (Meeusen et al., 2006). However, Meesters et al. found a significant association between the seasonal loss of energy and vegetarianism only in the Dutch dataset and not in the Finnish data. This might be because, in both datasets, the question about special diet adherence was based on self-reported questionnaires and could be subjected to misinterpretation. Additionally, since the patients retrospectively recalled the diet, recall bias may also have occurred.

Two studies attained inconsistent conclusions on the relationship between alcohol consumption and SAD (Krauchi and Wirz-Justice, 1988; Morales-Munoz et al., 2017). Such a disparity might be due to the different definitions of exposure (alcoholism vs. alcohol intake) and the different food questionnaires used in the studies. One systematic review and meta-analysis explored the link between AUD and major depression, showing that the presence of either disorder doubled the risks of the other disorder, with pooled, adjusted odds ratios ranging from 2.00 to 2.09 (Boden and Fergusson, 2011). Since AUD plays a critical role in the etiology of depression, considering the effects of alcohol abuse on individuals' social, economic, and legal circumstances, they further stated that AUD causing major depression was the most plausible causal association between AUD and major depression, not vice versa. However, it is entirely possible that alcohol is used as a coping mechanism for the development of depressive symptoms. Notably, Rosenthal et al. did not observe a significant association between alcohol intake and SAD. This was probably because they included participants with average alcohol intake at 4.4–5.0 dl/month and did not report any case of repetitive problems with alcohol at the social, interpersonal, legal, and occupational levels. Therefore, it is possible that normal alcohol consumption was not associated with SAD development, but hazardous alcohol intake imposes a negative impact on SAD.

It has been reported that atypical symptoms of depression, such as hyperphagia and weight gain, are frequently associated with SAD. Emotional eating has often been suggested to be one mechanism linking depression and subsequent development of obesity (Konttinen et al., 2019). Indeed, three included studies found that binge eating, restraint eating, emotional eating, and external eating were frequently observed in subjects with SAD symptoms (Berman et al., 1993; Krauchi et al., 1997; Donofry et al., 2014). Such data was in line with the report from a large cross-sectional study from 1,060 remitted depression patients, 309 currently depressed patients, and 381 healthy controls in the Netherlands. They found that remitted and current depressive disorders were significantly associated with more emotional eating (*p* < 0.001) and more external eating (*p* < 0.001) in a dose-response fashion (Paans et al., 2018), although Rosenthal et al. reported that eating style did not alter with a depressive state (SAD before and after light treatment) or seasonal variation (winter vs. summer). Furthermore, their finding that only emotional eating remains significant when adding external eating to the regression, but not the other way around, suggests that treatment against affect regulation problems, exemplified by emotional eating, and coaching patients to acquire better emotion regulation skills may possibly diminish SAD and its adverse health consequences.

To explore the efficacy of nutrition supplementation against SAD, we included two RCTs that gave SAD participants either vitamin D or vitamin B12 for potential SAD treatment, but neither of them showed significant beneficial effects in ameliorating SAD symptoms. In the country where the vitamin D supplementation RCT was performed, suboptimal 25-hydroxyvitamin D [25(OH)D] status is common (Frandsen et al., 2014). It has been anticipated that clinical trial participants with known symptoms of SAD would develop vitamin D insufficiency during wintertime and, thereby, benefit from vitamin D supplementation. One underlying reason why the study failed to observe a significant difference might be because the design did not favor inclusion of participants with lower 25(OH)D, so the study did not allow an investigation of the ability of vitamin D to improve mood in those with low 25(OH)D at baseline. In addition, this study selected a SPAQ-SAD cut-off of 8 to include more SAD participants, and such a score was less severe than that recommended by Kasper et al. (1989), which also reduced the possibility of observing a statistically significant difference. Nevertheless, such a result was in line with a large controlled clinical trial with 2,117 women that were given either 800 IU of vitamin D with 1,000 mg of calcium or placebo for 6 months. Results showed that the mental component score that reflected participants' subjective psychological well-being was not significantly different in the placebo group, indicating that supplementing women daily with a combination of vitamin D and calcium was not effective in promoting mental health or preventing wintertime blues (Dumville et al., 2006). Interestingly, another small RCT with 44 healthy subjects reported that giving subjects 400 or 800 IU of vitamin D3 per day significantly enhanced subjects' Positive Affect scores (enthusiastic, interested, and determined) but did not affect their Negative Affect scores (scared, afraid, and upset) (Lansdowne and Provost, 1998). One possible explanation for the disparity between this study and the previous two trials lies in the different vitamin D forms used as supplementation. Lansdowne et al. used vitamin D3, while the previous two studies employed vitamin D2. According to a meta-analysis of RCTs, supplementation with vitamin D3 imposes a significant and positive effect in the raising of serum 25(OH)D concentrations compared to the effect of vitamin D2, indicating that vitamin D3 is more efficacious and could potentially become the preferred choice for vitamin D supplementation (Tripkovic et al., 2012). We should also not neglect the fact that, in the study by Lansdowne et al., they only observed a significant improvement in participants' scores in Positive Affective, which are positive emotions, but the scores in Negative Affective remained unchanged. This raises the possibility that vitamin D supplementation is effective only in promoting optimistic feelings but not in alleviating negative emotions such as depression, especially in a population that does not have vitamin D deficiency.

Cobalamin, one of the active vitamin B_12_ forms, plays a key role in neural health (Thakkar and Billa, 2015; Li et al., 2016). A deficiency status of cobalamin would inhibit the physiological formation of the myelin sheath, subsequently altering correct nerve transmission (Rizzo et al., 2016) leading to impaired memory function and cognition and depression (Mikkelsen et al., 2016). However, in the study by Oren et al., the treatment with vitamin B_12_ was ineffective in SAD patients. This could also be due to the inclusion of participants with adequate vitamin B_12_ at baseline. Plasma vitamin B_12_ levels of the participants were 447 ± 164 pg/ml, which is within the reference range for the laboratory at 200–900 pg/ml. Another limitation of this study was the short interventional period. However, a 2-week trial of light therapy for SAD was proven to be sufficient and effective (Avery et al., 2001), which indicates that vitamin B_12_ supplementation might not be as effective as other treatment strategies, such as light therapy. Another factor that might lessen the effect of vitamin B_12_ supplementation to null was the large placebo effect observed in the study, which obligates the treatment effect to be very robust in order to show a statistical difference between placebo and intervention. Furthermore, it should be noted that the vitamins folic acid, B_12_, B_6_, and B_2_ jointly participate in one-carbon metabolism, in which methionine, from methyl-tetrahydrofolate reduction, is converted to S-adenosylmethionine, a universal donor of methyl groups, involving DNA, RNA, hormones, neurotransmitters, and others (Selhub, 2002). Because of these functions, one-carbon metabolism has drawn attention from researchers in preventing or treating diverse diseases including depression (Sugden, 2006; Assies and Pouwer, 2008; Baek et al., 2013; Assies et al., 2014; de Vries et al., 2015). Considering the synergistic effect of the vitamin B group in modulating biological and physiological responses, it warrants further investigation of how the vitamin B group, instead of single vitamin B_12_ supplementation, plays a role against SAD.

In the study by Krauchi et al., they raised the possibility that adjusting the percentage of macronutrients in SAD patients' diets may lead to certain therapeutic effects (Krauchi and Wirz-Justice, 1988). Nevertheless, the three RCTs that aimed to explore the impact of carbohydrate administration in SAD patients obtained different results (Rosenthal et al., 1989; Danilenko et al., 2008; Mischoulon et al., 2010). Results from Rosenthal's study were diverted between SAD patients and non-clinical subjects (Rosenthal et al., 1989). It has been hypothesized that the carbohydrate craving and increased carbohydrate consumption in SAD patients might be exerting their transient therapeutic effects by acting on serotonergic systems. However, SAD patients exhibited faster post-glucose glycemic and insulin responses and increased hedonic ratings of high concentrated sucrose solutions with depressive emotions than under euthymic situations (Krauchi et al., 1999). This might lead to a vicious cycle whereby the over-ingestion of carbohydrates may lower circulating glucose to subnormal levels after the surge of insulin, subsequently triggering elevated carbohydrate cravings and consumption. On the other hand, two other studies found no significant statistical differences in antidepressant effects between short-term carbohydrate or placebo in SAD patients (Danilenko et al., 2008; Mischoulon et al., 2010), although carbohydrate-heavy meals reduced evening hunger compared to the placebo meal (Mischoulon et al., 2010). These two studies are limited by small numbers of subjects and relatively short durations of intervention. With the combination of two independent studies, it also raises the possibility of dissimilar treatment conditions and ungeneralizable conclusions (Mischoulon et al., 2010).

Given the insufficient evidence showing beneficial efficacy of single nutrient supplementation or single diet intervention against SAD, it is entirely possible that providing only nutrient supplementation is not sufficient to mitigate the disease, while a combination of nutritional intervention, pharmacological therapy, and light therapy may augment the treating effect against SAD (Oren et al., 1994; Penders et al., 2016; Cools et al., 2018). Additionally, the RCTs included in this systematic review only provided SAD subjects with supplementation of a single nutrient or diet intervention with a fixed macronutrient percentage. Since the alteration of mood and sense of depression could be a consequence of several biological changes or resulting from various hormonal and nutritional cues (Kurlansik and Ibay, 2012), supplementation with a mix of nutrients will possibly lead to more beneficial results. In addition to vitamin B12 and vitamin D, anti-depression effects were observed in the controlled clinical trials with some other nutrients such as n-3 polyunsaturated fatty acids, zinc, and inositol (Mukai et al., 2014; Deacon et al., 2017; Schefft et al., 2017), which makes combination therapy of multiple nutrients, or interventions with dietary patterns and whole foods, seem logical, as they work in synergy with and function as the elements of the human biological network (Tapsell et al., 2016; Li et al., 2017, 2018).

However, it should be noted that there are several limitations to our current work. Only English-language papers were included in the current review, which limits our ability to incorporate evidence shown in other languages that might be relevant. In addition, this review only included published studies, which may have introduced publication bias since studies with negative results were less likely to be reported. The studies included in this systematic review were carried out using diverse methods (i.e., different SAD screening tools and different ways of performing statistical analysis), which raises challenges for homogenizing conclusions. Therefore, additional studies investigating diet, eating behavior, and nutrition intervention on SAD symptoms are warranted for a systematic review with more complexity and homogeneity.

## Conclusion

All RCTs reviewed in the current work are lacking in sample size or good study design. For observational studies, an enhanced strategy of statistical model building should be established. Additionally, results need to be replicated in double-blinded RCTs with larger populations and longer durations before any conclusions can be drawn about nutritional interventions.

## Author Contributions

YY and SZ performed literature search and data collection. XZ and YX performed the risk of bias assessment. YY, SZ, and JC conducted the study design and wrote the manuscript. XY provided scientific proofreading and supervised the study.

## Conflict of Interest

The authors declare that the research was conducted in the absence of any commercial or financial relationships that could be construed as a potential conflict of interest.
